# Comparison of potential long-term costs for preventive dentistry treatment of post-orthodontic labial versus lingual enamel cavitations and esthetically relevant white-spot lesions: a simulation study with different scenarios

**DOI:** 10.1186/s13005-019-0204-x

**Published:** 2019-08-09

**Authors:** Michael Knösel, Roberto Vogel Alvarez, Moritz Blanck-Lubarsch, Hans-Joachim Helms

**Affiliations:** 1 Department of Orthodontics, University Medical Center Göttingen, Göttingen, Germany; 2 Department of Orthodontics and Paediatric Dentistry, Universidad de La Frontera (UFRO), Temuco, Chile; 3 Private Practice, Hamburg, Germany; 4 Department of Orthodontics, University Hospital Münster, Münster, Germany; 5 Department of Medical Statistics, University Medical Center Göttingen, Göttingen, Germany

**Keywords:** Orthodontic treatment, Long-term costs, Decalcification, White spot lesion, Lingual treatment, Cost simulation

## Abstract

**Background:**

Post-orthodontic white-spot lesions (WSL) in esthetically relevant incisor and canine areas impair dentofacial esthetics, and preventive dentistry treatment is definitely required in case of enamel cavitations. The incidence of lingual post-orthodontic WSL and cavitation following lingual MB treatment has been reported to be distinctively decreased compared to labial MB treatment. Moreover, lingual WSL do not impair dentofacial esthetics. It was the objective of this study to calculate consequential costs of preventive dental care necessary to recover labial or lingual post-orthodontic cavitations as well as esthetically relevant WSL following either labial or lingual MB interventions.

**Methods:**

MB treatments (labial / lingual) were simulated in 1,000,000 patients between the ages of 12-18Y, with a median residual life time expectancy of 58Y based on local mortality tables. Range of MB Tx duration was 9–45 mo. Frequencies of post-orthodontic (labial / lingual) enamel damages were derived from large-scale WSL incidence studies. Anterior composite survival rates were based on a systematic review on the subject. Within the context of the German dental fee system (GOZ 2.3 and 3.5 fee increments), simulation of costs for enamel damage treatment and re-treatment (maximum: 5x) were based on single-surface composite restorations for lingual or labial cavitations and labial WSL treatment; and lingual WSL fluoridation.

**Results:**

Overall mean total costs for Tx and re-Tx of both WSLs and cavitations may sum up to 1718.91 Eur in the high-cost (GOZ 3.5) scenario for conventional MB cases, versus 19.94 Eur for lingually treated cases, given that renewal of simulated single-surface restorations takes place at 15-year intervals. When focussing on patients diagnosed with least of one WSL, and/or cavitation, these mean costs increase up to 2332.35 Eur for conventionally treated MB patients, or 65.03 Eur for lingual MB patients.

**Conclusion:**

Costs for repeated treatment of post-orthodontic enamel damages produced by conventional vestibular fixed appliances may easily exceed the initially higher costs associated with lingual orthodontic treatment. Judged economically in the long term, lingual MB Tx may be considered as a more cost-effective solution for a correction of malocclusion.

## Introduction

White-spot lesions (WSL) are a common undesired side-effect of multi-bracket (MB) treatment. Depending on the preventive strategy chosen, the incidence of WSL has mostly been reported to vary between 24 and 72.9%, with some reviews reporting post-orthodontic WSL occurrence of even 96% [1–3]. Well conducted studies with adequate numbers of subjects report a subject-related WSL incidence to range from 60.9 to 72.9%, depending on the assessed tooth group [2, 4]. While labial WSL in esthetically relevant incisor and canine areas impair dentofacial esthetics, preventive dentistry treatment is definitely required in case of enamel cavitations. According to a rough estimate of annual costs reported by Ren et al. [5], those biofilm-related enamel defects have recently been reported to accrue preventive dentistry treatment costs of up to 500,000,000 USD per year, relating to the proportion of orthodontically treated US citizens. However, on the one hand, this report seemed to be based on a more or less across-the-board cost estimate per patient, and transferring a lump sum of restorative treatment cost that was based on the dutch health care system to the situation of the US. On the other hand, composite restoration like all other dental health care interventions require repair, maintenance, or renewal in certain intervals [6], and those follow-up interventions accrue additional costs [7].

In view of the numbers of patients potentially affected by the problem of post-orthodontic WSL formation, it is of high interest for the specialty of orthodonics to find a viable solution to cope with the coincidences of (1) fixed orthodontic treatment, (2) a generally enhanced caries activity in (pre-) adolescents [1] and (3) teenager’s tendencies to neglect oral hygiene at this age.

Three-dimensionally controlled fixed orthodontic treatment may be performed using conventional labial attached MB appliances, or lingual MB appliances [2, 8, 9]. Whilst lingual MB treatment may be initially more laborious and costly due to external fabrication costs, the incidence of lingual post-orthodontic WSL and cavitation has been reported to be distinctively lower in terms of frequencies and in extent, in comparison to labial MB treatment [10–13]: Choosing lingual enamel areas for bracket placement that are known to be less susceptible to caries formation, the incidence of lingual post-orthodontic enamel decalcifications has been reported to be reduced up to the factor of ten for distinctive groups of teeth [12]. In a consecutively recruited sample of 385 subjects with a total of 10,162 trial teeth, a tooth-related WSL [cavitation] incidence of 5.8% [0.2%] in upper incisors, and a subject-related (i.e., subjects with at least one new WSL) incidence of 13.8% [0.8%] was reported. Considering complete upper and lower dental arches including first molars, there was a tooth-related WSL [cavitation] incidence of 2.4% [0.1%] [12]. As an additional positive side-effect, lingual WSL do not impair dentofacial esthetics, and may be adequately treated by local fluoridation, as inactive, remineralised lingual lesions do not require further treatment [14].

It is therefore the objective of this study to compare consequential costs with therapeutic preventive dentistry measures necessary to restore post-orthodontic enamel defects. The cost calculation and follow-up cost comparisons between labial and lingual MB interventions will address the dental rehabilitation on two levels:

*A,* Comprehensive rehabilitation of post-orthodontic enamel defects, including restoration of cavitations, and rehabilitation of esthetically relevant (visible by external view) white-spot lesions, as well as remineralisation / fluoridation of non-esthetically (not visible by external view) relevant white-spot lesions.

*B,* Minimum dental rehabilitation: restoration of cavitations, only.

## Method

In this cost calculation model, we simulated the treatment of post-orthodontic WSLs and –cavities, following either labial or lingual orthodontic MB interventions in 1,000,000 patients, based on and adopting the following parameters or reported evidence:

### WSL and caries incidence

Frequencies of post-orthodontic lingual or labial WSL and cavitations were derived from the two largest reports in the literature meeting the following criteria for each intervention type (lingual, labial MB) [2, 12].

Criteria for the choice of the reports used as calculation basis were

- fixed orthodontic treatment of complete upper and lower dental arches up to the first molars with either lingual or labial MB appliances
- no additional measures taken to reduce WSL incidence such as enamel sealants other than regular local home fluoridation
- enamel damage assessments of complete upper and lower dental arches up to the first molars
- adequate sample sizes of least of 350 subjects
- report of WSL and cavity incidence on both tooth- and patient level
- report of method error analyses

Incidences of post-orthodontic WSLs and cavitations per month were calculated by:

$$ \left(\mathrm{total}\ \mathrm{reported}\ \mathrm{WSL}\ \mathrm{or}\ \mathrm{cavitation}\ \mathrm{frequencies}/\mathrm{number}\ \mathrm{of}\ \mathrm{patients}\right)/\mathrm{mean}\ \mathrm{treatment}\ \mathrm{duration} $$

The dispersion parameter was chosen in a way to reflect percentages of patients without occurrence of a WSL or caries, based on the cited reports [2, 12] (break-down to single months, Table 1).

**Table 1:** Subject sample and treatment characteristics

Orthodontic intervention: MB	Lingual	Labial	Lingual	Labial	Lingual	Labial	Lingual	Labial
Enamel defect interventions: 1, *Fluoridation of WSL;* 2, *single surface restoration of WSL;* 3, *single surface restoration of cavitation*	1 + 3	2 + 3	1 + 3	2 + 3	1 + 3	2 + 3	1 + 3	2 + 3
number of generated patients [n_sim_]	1000000	1000000	1000000	1000000	1000000	1000000	1000000	1000000
max. Number of teeth 16–46	24	24	24	24	24	24	24	24
minimum age at treatment start (years)	12	12	12	12	12	12	12	12
maximum age at treatment start (years)	18	18	18	18	18	18	18	18
median time until death (mo)	696	696	696	696	696	696	696	696
maximum age (years)	100	100	100	100	100	100	100	100
minimum bracket treatment duration (month)	9	9	9	9	9	9	9	9
maximum bracket treatment duration (mo)	45	45	45	45	45	45	45	45
WSL incidence rate per month^a^	0.0248	0.1537	0. 0248	0.1537	0. 0248	0.1537	0. 0248	0.1537
Cavitation incidence rate per month^a^	0.0012	0.0015	0. 0012	0.0015	0. 0012	0.0015	0. 0012	0.0015
Dispersion parameter WSL [dis_WSL_]	0.3	0.72	0.3	0.72	0.3	0.72	0.3	0.72
Dispersion parameter cavitation [dis_CAV_]	1	1	1	1	1	1	1	1

^a^Incidence rates are based on previous reports by Knösel et al. (2015)*,* and Richter et al. (2011) [2, 12]

### Orthodontic intervention simulation

The following steps were repeated n_sim_ = 1,000,000 times, using the software R (http://www.r-project.org, v 3.1.1):

- Subject’s age at initiation of MB treatment (t0) was generated by using a uniform distribution with boundaries of a minimum age of 12 years, and a maximum age of 18 years (age = U(12,18)).
- Labial or lingual MB treatment duration (i. e., time elapse between initiation of MB (t0) and debonding) was modeled as having a uniform distribution for both interventions, and set within the boundaries reported in the literature [12], with a minimum of (min.bra.time) 9 months and a maximum (max.bra.time) of 45 months (treatment duration = U(9,45)).

### WSL and caries formation simulation

Frequencies of WSLs and cavitations were generated using a negative binomial distribution with the corresponding incidence rates (per month) and dispersion parameter. The incidence rate was multiplied by the treatment duration to account for the higher risk of WSL or caries formation during longer treatment durations. The maximum number of affected teeth per patient was set to *n* = 24.

### Subject’s residual lifetime calculation

Subject’s remaining median lifetime expectancy (i. e., time elapse between MB debonding, and subject’s death) of 696 months (58 years) was simulated on the basis of official German mortality tables (− 20 years) [15], and modeled using a Weibull distribution with shape parameter = 1. The theoretical maximum age (age at t0 + treatment duration + survival time) was set to 100 years.

### WSL and caries treatment, re-treatment and follow-up or repair simulation

Lingual or labial cavitations and labial WSL treatment was simulated by single-surface composite restorations, while lingual WSL treatment was simulated by a single local fluoride application. Anterior composite resoration survival rates were derived from the findings of a recent systematic review of the literature on the subject [6]: As two relevant articles were identified [16, 17], indicating two different composite restoration survival times, and in order to address both scenarios, median time until re-treatment or repair was calculated for different scenarios:

- 50% failure after 15 years median time (average survival time 15 years; med.car.time = 15*12 months) [16]

and

- 80% survival rate at 10 years (average survival time 31 years; lambda = −log(0.8)/10; med.car.time = −log(0.5)/lambda; med.car.time = 31*12 month) [17].
- In addition to the future cost simulation based on composite survival rates of 15 or 31 years, the same cost calculations were repeated with a reduced median time until need for repair (i. e., 5 years / 60 months, or 10 years / 120 months, respectively), in order to give an idea of expected costs for shorter repair intervals for composite restorations.

For each observed and treatment-simulated WSL or caries, the time until the next repair or re-treatment was calculated using, again, a Weibull distribution with shape parameter 1 and median time until a repair is needed (med.ws.time; med.car.time). If the time to re-treatment or repair of composite restorations undercut the subject’s survival time, another repair was calculated and above calculation were repeated, until a maximum of *n* = 5 repairs or re-restorations was met.

### WSL and caries treatment and re-treatment cost calculation

Costs were calculated in Euro (Eur). Within the context of the German dental fee system, we compared the intervention of a single-surface anterior or buccal composite restoration or re-restoration, or fluoridation, with GOZ (Gebührenordnung für Zahnärzte / Catalogue of Fees) [18] increment factors of 2.3, or 3.5; Table 2 a and b give details on the costs for the different interventions.

**Table 2:** a and b Dental fees for local fluoridation, and single surface composite restorations or re-restorations based on the German Private Dental Fee Catalogue GOZ. In this model, a private-payer perspective was adopted, as single-surface composite restorations are mostly subject to private payment by the patient. Calculations were based on both fee increment factors of 2.3 and 3.5, as factoring of chargeable item-points is common to determine fees of private dental treatment in Germany

Treatment	Dental fee position (GOZ)	Fee increment factor 2.3 (Eur)	Fee increment factor 3.5 (Eur)
a. Fluoridation, one single application, per application / session.			
Topical fluoridation	1020	6.47	9.84
Total		6.47	9.84
Treatment	Dental fee position (GOZ)	Fee increment factor 2.3 (Eur)	Fee increment factor 3.5 (Eur)
b. Dental fees for single surface composite restoration or re-restoration, per surface. Not included were costs for anaesthesia (position 40/41a, 11.84 Eur) or radiographic assessments.			
Specific measures during preparation or filling	2030	8.41	12.80
Use of rubberdam	2040	8.41	12.80
Adhesive restoration, one surface	2060	68.17	103.74
Total		84.99	129.34

Following these simulation steps, the costs were derived by multiplying the costs per WSL (caries) situation with the number of WSL (caries) situations observed. The repair or re-restoration costs were derived by multiplying the costs per WSL (caries) situation with the total number of repairs done.

## Results

The results of the cost calculation for simulated initial treatment and re-treatment of lingual or labial cavitations and WSL are displayed in detail in Tables 3 and 4, separately for enamel defects following fixed orthodontic labial or lingual treatment, complete rehabilitation of all enamel damages or cavitations only, survival rates of 15 or 31 years, and dental fee increment factors (GOZ) 2.3 or 3.5. Within the limitations set by the model parameters, and considering all patients with or without enamel defects, long-term costs for preventive dentistry treatments following labial [lingual] MB interventions amount up to an average 1718.91 [19.94] Eur in the case of a 15Y-survival rate and factor 3.5 cost calculations. Considering patients with the diagnosis of least of one cavitation, separately, those costs would rise to an average 2361.34 [444.20] Eur within this subject group.

**Table 3:** Cost calculation based on the German Private Dental Fee Catalogue, for simulated initial treatment and re-treatment of lingual or labial cavitations and WSL, separately for enamel defects following labial or lingual MB treatment, complete rehabilitation of all enamel damages or cavitations only, survival rates of 15 or 31 years, and dental fee increment factors (GOZ) 2.3 or 3.5. All costs are given in Eur

Orthodontic intervention: MB	Lingual	Labial	Lingual	Labial	Lingual	Labial	Lingual	Labial
Enamel defect interventions: 1, *Fluoridation of WSL;* 2, *single surface restoration of WSL;* 3, *single surface restoration of cavitation*	1 + 3	2 + 3	1 + 3	2 + 3	1 + 3	2 + 3	1 + 3	2 + 3
median time until composite restoration repair (mo)	180	180	180	180	372	372	372	372
maximum number of composite re-treatments per tooth	5	5	5	5	5	5	5	5
Dental fee increment factors (GOZ, 2.3 or 3.5)	2.3	2.3	3.5	3.5	2.3	2.3	3.5	3.5
Treatment costs for one post-orthodontic WSL	6.47	84.99	9.84	129.34	6.47	84.99	9.84	129.34
Treatment costs for one post-orthodontic cavitation	84.99	84.99	129.34	129.34	84.99	84.99	129.34	129.34
Re-treatment (follow-up) costs for one post-orthodontic WSL, per cycle	0	84.99	0	129.34	0	84.99	0	129.34
Re-treatment (follow-up) costs for one post-orthodontic cavitation, per cycle	84.99	84.99	129.34	129.34	84.99	84.99	129.34	129.34
Number of patients with costs (at least one WSL and/or cavitation) [costs.pop1]	307839	736211	306613	736988	307930	736155	306626	736421
Number of patients with costs for caries (at least one cavitation, patients could have WSL in addtion) [costs.pop2]	30491	38116	30550	38349	30726	37922	30556	38519
Percentage of costs.pop1 subgroup of all simulated patients (costs.pop1/nsim)	0.308	0.736	0.307	0.737	0.308	0.736	0.307	0.736
Percentage of costs.pop2 subgroup of all simulated patients (costs.pop2/nsim)	0.031	0.038	0.031	0.038	0.031	0.038	0.031	0.039
mean number of WSLs, per patient	0.669	4.023	0.671	4.029	0.671	4.026	0.669	4.03
Number of patients with at least one WSL	286817	726844	285463	727577	286775	726942	285470	726989
mean number of cavitations, per patient	0.0316	0.0399	0.0316	0.0402	0.0319	0.0397	0.0317	0.0403
Number of patients with at least one cavitation	30491	38116	30550	38349	30726	37922	30556	38519
mean initial WSL Tx costs following de-bracketing, per patient	4.33	341.91	6.60	521.05	4.34	342.14	6.58	521.23
mean initial cavitation Tx costs following de-bracketing, per patient	2.68	3.39	4.09	5.19	2.71	3.37	4.10	5.21
mean WSL re-treatment (follow-up) costs, per patient	0.00	775.21	0.00	1180.95	0.00	399.45	0.00	606.74
mean cavitation re-treatment (follow-up) costs, per patient	6.06	7.67	9.25	11.72	3.16	3.92	4.79	6.02
mean total WSL costs (initial Tx + Re-Tx (follow-up) costs)	4.33	1117.12	6.60	1702.00	4.34	741.59	6.58	1127.98
mean total cavitation costs (initial Tx + Re-Tx (follow-up) costs)	8.74	11.06	13.34	16.91	5.86	7.30	8.90	11.23
**mean total (WSL + cavitation initial Tx + Re-Tx) costs, for all subjects including non-affected patients, per patient**	**13.07**	**1128.17**	**19.94**	**1718.91**	**10.21**	**748.89**	**15.48**	**1139.21**
mean initial WSL Tx costs following de-bracketing, per patient, for patients with at least 1 WSL and/or cavitation	14.06	464.41	21.52	707.00	14.10	464.76	21.46	707.79
mean initial cavitation Tx costs following de-bracketing, per patient for patients with at least 1 WSL and/or cavitation	8.72	4.60	13.35	7.05	8.80	4.58	13.38	7.07
mean WSL Re-Tx (follow-up) costs following de-bracketing, per patient, for patients with at least 1 WSL and/or cavitation	0.00	1052.98	0.00	1602.40	0.00	542.62	0.00	823.91
mean cavitation Re-Tx (follow-up) costs following de-bracketing, per patient, for patients with at least 1 WSL and/or cavitation	19.69	10.41	30.16	15.90	10.25	5.33	15.63	8.18
mean total WSL (Tx + Re-Tx) costs following de-bracketing, per patient, for patients with at least one WSL and/or cavitation	14.06	1517.39	21.52	2309.40	14.10	1007.38	21.46	1531.70
mean total cavitation (Tx + Re-Tx) costs following de-bracketing, per patient, for patients with at least 1 WSL and/or cavitation	28.40	15.02	43.51	22.95	19.04	9.91	29.01	15.25
**mean total (WSL + cavitation initial Tx + Re-Tx) costs, per patient, restricted to patients with at least 1 WSL and/or cavitation**	**42.47**	**1532.41**	**65.03**	**2332.35**	**33.14**	**1017.30**	**50.47**	**1546.96**
mean initial WSL Tx costs following de-bracketing, per patient, for patients with at least 1 cavitation	4.99	388.27	7.48	590.45	4.99	391.17	7.55	590.17
mean initial cavitation Tx costs following de-bracketing, per patient, for patients with at least 1cavitation	87.99	88.88	133.97	135.43	88.15	88.95	134.24	135.23
mean WSL Re-Tx (follow-up) costs following de-bracketing, per patient, for patients with at least one cavitation	0.00	875.79	0.00	1329.83	0.00	459.85	0.00	689.06
mean cavitation Re-Tx (follow-up) costs following de-bracketing, per patient, for patients with at least 1 cavitation	198.78	201.17	302.75	305.63	102.69	103.49	156.89	156.38
mean total WSL (Tx + Re-Tx) costs following de-bracketing, per patient, for patients with at least 1 cavitation	4.99	1264.06	7.48	1920.28	4.99	851.02	7.55	1279.23
mean total cavitation (Tx + Re-Tx) costs following de-bracketing, per patient, for patients with at least 1 cavitation	286.77	290.04	436.72	441.07	190.84	192.44	291.13	291.61
**mean total (WSL + cavitation initial Tx + Re-Tx) costs, per patient, restricted to patients with at least 1 cavitation**	**291.75**	**1554.11**	**444.20**	**2361.34**	**195.83**	**1043.46**	**298.68**	**1570.84**

**Table 4:** In addition to the cost calculation based on composite survival rates of 15 or 31 years (Table 3), the same cost calculations were repeated with a reduced median time until need for repair (i. e., 5 years / 60 months, or 10 years / 120 months, respectively), in order to give an idea of expected costs for shorter repair intervals for composite restorations. Other than that, identical settings of dental fee increment factors (GOZ 2.3, or 3.5) and treatment fees (in Eur) have been used

Orthodontic intervention: MB	Lingual	Labial	Lingual	Labial	Lingual	Labial	Lingual	Labial
Enamel defect interventions: 1, *Fluoridation of WSL;* 2, *single surface restoration of WSL;* 3, *single surface restoration of cavitation*	1 + 3	2 + 3	1 + 3	2 + 3	1 + 3	2 + 3	1 + 3	2 + 3
median time until composite restoration repair (mo)	60	60	60	60	120	120	120	120
maximum number of composite re-treatments per tooth	5	5	5	5	5	5	5	5
Dental fee increment factors (GOZ, 2.3 or 3.5)	2.3	2.3	3.5	3.5	2.3	2.3	3.5	3.5
Treatment costs for one post-orthodontic WSL	6.47	84.99	9.84	129.34	6.47	84.99	9.84	129.34
Treatment costs for one post-orthodontic cavitation	84.99	84.99	129.34	129.34	84.99	84.99	129.34	129.34
Re-treatment (follow-up) costs for one post-orthodontic WSL, per cycle	0	84.99	0	129.34	0	84.99	0	129.34
Re-treatment (follow-up) costs for one post-orthodontic cavitation, per cycle	84.99	84.99	129.34	129.34	84.99	84.99	129.34	129.34
**mean total (WSL + cavitation initial Tx + Re-Tx) costs, for all subjects including non-affected patients, per patient**	**17.76**	**1702.21**	**26.95**	**2594.50**	**15.13**	**1386.09**	**23.03**	**2102.93**
mean initial WSL Tx costs following de-bracketing, per patient, for patients with at least 1 WSL and/or cavitation	14.12	464.36	21.39	707.98	14.04	464.74	21.48	706.50
mean initial cavitation Tx costs following de-bracketing, per patient for patients with at least 1 WSL and/or cavitation	8.83	4.64	13.44	7.05	8.78	4.57	13.36	7.04
mean WSL Re-Tx (follow-up) costs following de-bracketing, per patient, for patients with at least 1 WSL and/or cavitation	0.00	1822.67	0.00	2777.64	0.00	1397.78	0.00	2121.42
mean cavitation Re-Tx (follow-up) costs following de-bracketing, per patient, for patients with at least 1 WSL and/or cavitation	34.78	18.26	52.71	27.62	26.44	13.74	40.15	21.03
mean total WSL (Tx + Re-Tx) costs following de-bracketing, per patient, for patients with at least one WSL and/or cavitation	14.12	2287.04	21.39	3485.62	14.04	1862.52	21.48	2827.91
mean total cavitation (Tx + Re-Tx) costs following de-bracketing, per patient, for patients with at least 1 WSL and/or cavitation	43.61	22.90	66.15	34.67	35.22	18.31	53.51	28.07
**mean total (WSL + cavitation initial Tx + Re-Tx) costs, per patient, restricted to patients with at least 1 WSL and/or cavitation**	**57.72**	**2309.93**	**87.54**	**3520.29**	**49.27**	**1880.83**	**74.99**	**2855.98**
mean initial WSL Tx costs following de-bracketing, per patient, for patients with at least 1 cavitation	5.00	388.42	7.47	596.19	4.91	389.06	7.62	597.26
mean initial cavitation Tx costs following de-bracketing, per patient, for patients with at least 1cavitation	88.13	88.99	133.86	135.06	88.07	88.97	134.03	134.99
mean WSL Re-Tx (follow-up) costs following de-bracketing, per patient, for patients with at least one cavitation	0.00	1527.71	0.00	2338.34	0.00	1168.57	0.00	1792.53
mean cavitation Re-Tx (follow-up) costs following de-bracketing, per patient, for patients with at least 1 cavitation	347.15	349.98	525.19	529.44	265.21	267.83	402.81	403.32
mean total WSL (Tx + Re-Tx) costs following de-bracketing, per patient, for patients with at least 1 cavitation	5.00	1916.13	7.47	2934.53	4.91	1557.63	7.62	2389.80
mean total cavitation (Tx + Re-Tx) costs following de-bracketing, per patient, for patients with at least 1 cavitation	435.28	438.96	659.05	664.50	353.28	356.79	536.84	538.31
**mean total (WSL + cavitation initial Tx + Re-Tx) costs, per patient, restricted to patients with at least 1 cavitation**	**440.28**	**2355.09**	**666.52**	**3599.03**	**358.19**	**1914.42**	**544.46**	**2928.10**

## Discussion

The formation of enamel decalcifications and / or cavitations is a frequent and undesired side-effect of conventional fixed orthodontic treatment, and one that has been reported to accrue follow-up costs of 500,000,000 USD per year in the USA [5]: Of an estimated number of 3,000,000 US-American patients with post-orthodontic WSL, up to 750,000 were assumed by those authors to require professional care [5]. However, those costs calculated by Ren et al. were rather based on a simple ballpark figure of potential costs, estimating fixed average costs of at least 650 USD for every patient with WSL, based on the dutch dental fee system, and transferring those dental fees to the assumed numbers of patients treated in the US [5].

It was the aim of our study to provide a more refined and more detailed follow-up cost calculation for labial WSLs, and also to calculate whether the use of lingual appliances may be suitable for reducing the extent of those follow-up costs, by choosing enamel areas for bracket placement that are known to be less susceptible to caries formation [10–13].

Our calculation yields the result that overall mean total costs for Tx and re-Tx of both WSLs and cavitations may sum up to 1718.91 Eur for conventional MB cases, versus 19.94 Eur for lingually treated cases, given that renewal of simulated single-surface restorations takes place at 15-year intervals. When focussing on the group of patients with at least 1 WSL and/or cavitation, these mean costs increase up to 2332.35 Eur for conventionally treated MB patients, or 65.03 Eur for lingual MB patients. Tables 3 and 4 display the future cost estimates for different composite renewal intervals, and regarding high-cost scenarios for conventional MB cases and patients with at least one new WSL or cavitation, these costs may vary between 1570.84 Eur (renewal interval of 31 yrs) and 3520.29 Eur (renewal interval of 5 years). That is, follow-up costs for repeated treatment of enamel damages set by orthodontic treatment using conventional appliances may easily reach or exceed the initially higher costs common in lingual orthodontic treatment. The costs in our simulation were based on the German Private Dental Fee Catalogue [18]. Calculated follow-up Tx costs may be subject to international variation based on local dental fee systems.

However, what has not been taken into detailed consideration here is the individual demand of the patient in terms of quality of enamel rehabilitation or restoration, as well as its frequency of renewal. While some may not feel bothered by WSLs in the visible area at all, others may be much more demanding in terms of their dentofacial appearance and are likely to increase the frequency the estimated composite or restoration renewal (due to e.g. composite discoloration) compared to the 15- or 31-year-intervals as suggested by the systematic review used as basis for calculation in our simulation [6, 17]. In order to address this situation and to give an estimate of costs for shorter intervals of composite renewal, cost simulations were repeated with a reduced median time until need for repair (i. e., 5 years, or 10 years, respectively). Future costs derived from this simulation exceed those based on longer renewal intervals distinctively and are displayed in Table 4.

Due to the similar frequencies of cavitations in lingually or conventionally treated cases (lingual: 0.0012/mo; labial: 0.0015/mo), costs and follow-up costs for cavitation treatment only are quite similar. However, it needs to be taken into account that the study by Richter et al. reports in detail both vestibular post-orthodontic enamel damages in terms of WSL (73%) and cavitations (2.3%); but interestingly, they also report an additional percentage of almost 5% of new vestibular restorations that have been made during fixed orthodontic treatment [2]. It is highly likely that those restorations were made due to formation of cavitation during MB treatment, and may be added to the incidence of post-orthodontic cavitations.

### Cost simulation limitations

Our model did not account for:

- Other related problems such as periodontal complications were not simulated by our model, especially as large-scale research on the subject of long-term periodontal problems triggered by fixed orthodontic treatments are few and far between.
- The additional laboratory costs that are common in fully customised lingual bracket treatment were not calculated, because these may vary between manufacturers, countries, and frequencies of ordering appliances by the clinician.
- Due to the large international variation in lingual or labial appliance costs and presence or absence of insurance coverage of direct, labial bonding or indirect bonding of lingual systems, appliance costs were not included in the calculation.
- Also, it is common that lingual treatments are usually charged higher than conventional MB treatment, as different clinicians are free to use different fee increments for their efforts.

### Discounting of future costs vs. increasing spending capacities and increasing dental fees

Some previous studies on long-term cost calculations of dental treatments discounted future costs by an annual 3%, in order to solve the problem that costs and long-term benefit of e.g. a composite restoration are not referring to the same time point: They used the method of discounting to refer the value of fees at different time points to the same time point [7, 19]. For example, re-restoration costs of 100 Eur due at 2 years following composite restoration treatment and 8 years of survival follow-up (after 10 years from t0) would be discounted to: 100/(1.03)^10 = 74.41 Eur at t0. It has to be considered that the level of the discounting percentage is often chosen more or less arbitrarily, with subsequent need for sensitivity analyses in order to analyze the robustness of the method in terms of the current cost calculation [19]. However, as a matter of fact, there is also a dynamic development of increasing monetary value and spending capacities, and also increasing dental fees, with a subsequent need to offset those additional costs with a potential discounting. Hence, neither methods for a discounting of dental fees, nor for an assumable increase or dynamic development of dental fees were implemented by our cost simulation.

### Critical consideration of factors influencing incidences of WSL

The present model simulates a very high sample size of 1,000,000 orthodontic treatments in order to achieve robust cost calculations, but it also necessarily simplifies some potential factors that may have an influence on WSL or cavitation treatment costs: Labial or lingual enamel decalcification or cavitation incidence rates per month were based on two of the largest reports on the subject, with adequate sample sizes of least of 350 patients [2, 12]. Previous reports on lingual post-orthodontic lesions yielded slightly decreased numbers of WSL compared to the report used here but were also smaller in sample size [10, 11, 13]. Many of the reports on labial post-orthodontic lesions also did not assess decalcifications in complete dental arches, but in specific tooth groups, such as upper incisors only [4]. In contrast, the reports we based our simulation on met the criteria of both full orthodontic treatment and WSL assessment of complete upper and lower dental arches. We did not consider results of studies including the use of additional prophylactic treatments that may be suitable for a further decrease of WSL frequencies (i.e., bracket sealants, professional cleaning sessions, mouth rinses): On the one hand, these measures are producing additional costs; on the other hand, their effectivness is highly dependent from factors that cannot be easily controlled and calculated, such as frequencies of cleaning sessions, or costs and durabilty of bracket sealants. It has been shown that the surface integrity of enamel sealants may be limited to an average of 3.5 months, with the subsequent necessity of efforts in terms of time and costs to maintain sealant layers throughout fixed orthodontic treatment, thereby resulting in additional follow-up prophylaxis costs [20, 21].

An increased susceptibility for caries formation in has been reported by a majority of studies on post-orthodontic enamel damages [1, 13]. Our model is comparable in terms of age-related caries activity given that both studies used as basis for our simulation have excluded patients of an age > 18 years at the time point of the start of treatment. In our cost simulation, start of MB interventions were therefore set at an age range of 12–18 years, while subject’s residual life time was calculated based on local mortality tables [15].

MB-Tx duration was calculated after parameters reported for lingual appliance treatment [12], and set to an identical, standardized range from 9 to 45 months for both labial and lingual MB interventions. The treatment duration in the two reference studies used here [2, 12] was 9–45 months in the case of lingual treatment, while in the conventionally bonded reference study the treatment duration of the sample was given in blocks, with roughly ¼ of treatment durations each under 22; 22–27; 27–33; and + 33 months [2]. In order to be able to compare both treatments without bias, the simulated treatment time was set to 9–45 months, thereby providing a ‚mixed calculation’ of short and longer interventions: To generate the individual treatment times per patient in a robust and consistent way, a univariate distribution U(9,45) was used to select a treatment time randomly within the interval from 9 to 45 month. The expected value as well as the Median of this distribution would be 27 months, meaning that approximately 50% of all simulated cases had a treatment duration of less than 27 months. With this approach treatment time variations (longer treatment durations yield a higher risk of WSL incidence) can be taken into account while keeping the average time around 27 months, thereby averaging out the impact of longer and shorter treatment durations when summarized across the simulated patients.

### Consideration of variation in restoration survival rates

Composite restoration survival rate was reported as being 80% after 10 years (average 372 Mo or 31 years) [17], or 50% after 15 years median time; (average 180 mo or 15 years) [16]. In order to address both scenarios, calculations were performed two-fold, based on the two different survival rates reported. However, the need for renewal of restorations for aesthetic reasons was not considered in the two studies used in our simulation, but only the need for re-restoration due to caries, fractures, and replaced restorations.

### Simplification of Tx to one-surface-restoration vs. hard tissue loss and non-treatment option

Lingual or vestibular cavitation treatment as well as labial WSL treatment was simulated by single-surface composite restorations, while lingual WSL received fluoridation. Costs for composite re-restorations, were simulated by a renewal of restorations with, again, single-surface composite fillings, up to 5 times per subject’s life time. However, repeated renewal of a restoration results in increasing loss of hard tissue, and, in turn, requiring more extensive restorations with every additional step of re-treatment [7]. This would have a deteorating effect on costs in patients with restorations; e.g., the dental fee position for a two-surface composite restoration instead of a single-surface restoration (GOZ fee position 2080 instead of 2060) would increase from 68.17 to 71.92 Eur at fee increment factor 2.3, or from 103.74 to 109.45 Eur at fee increment factor 3.5. In more demanding patients, restorations by veneers or crowns may apply, resulting in even higher costs.

The simulation of a standardized one-surface-restoration instead of partial use of multiple-surface composites, veneers, crowns or the option of non-treatment is to be seen as an averaging approach in the setting of this study. While it is obvious that a single-surface composite restoration may outrun the standard treatment of a labial WSL economically, with many WSL remaining untreated, other lesions may receive esthetically orientated care, such as resin infiltration, abrasion, composite restoration, or even veneers. Our simulation aims at offsetting potential costs for both increasing loss of hard tissues, with subsequent need for re-treatment, and variation in patient’s individual demands: The reason for simulating costs by a rather simple and repetitive treatment of WSL was to raise awareness and to give an estimate for potential follow-up costs, in an attempt to approximate or to compensate for factors that may lower (use of alternative treatments like micro-abrasion or partial or complete non-treatment of WSLs) or raise total costs (compensation for increasing hard-tissue loss with every re-treatment, with subsequent need for more extensive composite restorations, veneers, crowns etc. [7].

For the same reason, dental fee positions for anaesthesia, sensivity testing, and radiographic assessments as common with cavitation Tx care were not simulated in this model.

### Alternative WSL and cavitation treatment options

WSLs generate follow-up treatment costs, based on the assumption that the aim is to achieve a *restitutio ad integrum*. Costs may vary depending on patient’s personal esthetic demands, type and quality of WSL treatment, and subsequent oral hygiene. While treatment of lingual or labial side cavities is mandatory, treatment of inactive WSL is not, but often required by patients when occurring in esthetic relevant visible labial enamel areas. Inactive lingual WSL are not an esthetic problem, and are commonly not treated other than by local fluoridation / remineralisation.

No treatment or only partial treatment would be an option, i. e. restricting WSL treatment to e.g. upper and lower incisors or canines. To give an idea about the variation in costs, we calculated scenarios for both care for cavities, only, and cavity plus WSL treatment (Table 3).

There are alternatives to single-surface composite restorations of WSL for treating esthetically relevant enamel areas, such as micro-invasive infiltration. However, no long-term assessments of the esthetic effect of WSL infiltration exceeding 1 year observation are currently available [22, 23]. Moreover, the initial treatment of WSLs by infiltration may be increased compared to composite restorations, due to the higher costs of the infiltrant material [7].

Another alternative treatment would be the method of enamel / WSL micro-abrasion using hydrochloric acid / pumice slurries. However, also micro-abrasion is recommended to be used clinically with caution, as it is an invasive method that reduces markedly the enamel surface thickness [24], and one with a questionable stability of the achieved esthetic effects [25].

Any enamel changes induced by invasive methods are likely to be followed by a more extensive restorative measures at some point in time [7], thereby resulting in re-treatment costs that may be distinctively increased compared to the repeated single-surface composite restoration calculated here.

## Conclusions

Within the limitation of the averaging treatment simulation of a standardized one-surface-restoration instead of partial use of multiple-surface composites, resin infiltration, abrasion, composite restoration, veneers, crowns or the option of non-treatment, the following conclusions are drawn:

- The use of lingual appliances reduces the extent of post-orthodontic enamel damage and long-term follow-up costs in comparison to those caused by vestibular fixed orthodontic treatment.
- Orthodontic patients should be aware of and informed that costs for repeated treatment of enamel damages produced during fixed orthodontic treatment using conventional vestibular appliances may easily reach or exceed the initially higher costs associated with lingual orthodontic treatment.
- Judged economically in the long term, lingual MB Tx may be considered as a more cost-effective solution for a correction of malocclusion.

## Data Availability

All data generated or analysed during this study are included in this published article.
